# Association between subchondral bone structure and osteoarthritis histopathological grade

**DOI:** 10.1002/jor.23312

**Published:** 2016-06-22

**Authors:** Mikko A. J. Finnilä, Jérôme Thevenot, Olli‐Matti Aho, Virpi Tiitu, Jari Rautiainen, Sami Kauppinen, Miika T. Nieminen, Kenneth Pritzker, Maarit Valkealahti, Petri Lehenkari, Simo Saarakkala

**Affiliations:** ^1^ Research Unit of Medical Imaging, Physics and TechnologyFaculty of Medicine, University of OuluOuluFinland; ^2^ Medical Research Center OuluOulu University Hospital and University of OuluOuluFinland; ^3^ Department of Applied PhysicsUniversity of Eastern FinlandKuopioFinland; ^4^ Cancer and Translational Medicine Research UnitFaculty of MedicineUniversity of OuluOuluFinland; ^5^ Institute of Biomedicine, AnatomyUniversity of Eastern FinlandKuopioFinland; ^6^ Department of Diagnostic RadiologyOulu University HospitalOuluFinland; ^7^ Department of Laboratory Medicine and PathobiologyUniversity of Toronto and Mount Sinai HospitalTorontoOntarioCanada; ^8^ Department of SurgeryOulu University HospitalOuluFinland

**Keywords:** osteoarthritis, bone sclerosis, OARSI grading, micro‐computed tomography, histomorphometry

## Abstract

Despite increasing evidence that subchondral bone contributes to osteoarthritis (OA) pathogenesis, little is known about local changes in bone structure compared to cartilage degeneration. This study linked structural adaptation of subchondral bone with histological OA grade. Twenty‐five osteochondral samples of macroscopically different degeneration were prepared from tibiae of 14 patients. Samples were scanned with micro‐computed tomography (μCT) and both conventional structural parameters and novel 3D parameters based on local patterns were analyzed from the subchondral plate and trabecular bone. Subsequently, samples were processed for histology and evaluated for OARSI grade. Each bone parameter and OARSI grade was compared to assess structural adaptation of bone with OA severity. In addition, thicknesses of cartilage, calcified cartilage, and subchondral plate were analyzed from histological sections and compared with subchondral bone plate thickness from μCT. With increasing OARSI grade, the subchondral plate became thicker along with decreased specific bone surface, while there was no change in tissue mineral density. Histological analysis showed that subchondral plate thickness from μCT also includes calcified cartilage. Entropy of local patterns increased with OA severity, reflecting higher tissue heterogeneity. In the trabecular compartment, bone volume fraction and both trabecular thickness and number increased with OARSI grade while trabecular separation and structure model index decreased. Also, elevation of local patterns became longitudinal in the subchondral plate and axial transverse in trabecular bone with increasing OARSI grade. This study demonstrates the possibility of radiological assessment of OA severity by structural analysis of bone. © 2016 The Authors. *Journal of Orthopaedic Research* Published by Wiley Periodicals, Inc. J Orthop Res 35:785–792, 2017.

In osteoarthritis (OA), subchondral bone becomes sclerotic and undergoes structural changes such as the formation of osteophytes and bone cysts.^1^ It has been suggested that first tissue‐level changes in OA would occur in the subchondral bone before any signs of degeneration in the overlying articular cartilage,^2^, ^3^ and thus contribute to the pathogenesis of OA.^4^, ^5^, ^6^, ^7^ In respective of increasing evidence of subchondral bone modifications with OA, these changes are generally not assessed until late OA stages with clinical diagnostics and histopathology.

Recently, it has been suggested that different OA phenotypes (post‐traumatic, metabolic, ageing, genetic, pain) should be considered when studying OA and developing potential treatment or intervention.^8^ Most of the animal models studied are post‐traumatic and have shown a loss of bone in the trabecular compartment^9^, ^10^, ^11^, ^12^, ^13^, ^14^ and the subchondral plate,^12^, ^13^, ^14^, ^15^ whereas a thickening of the plate after trauma has also been reported.^9^ The loss of the subchondral plate has been associated with increased thickness of calcified cartilage.^14^ Most relevant models for ageing OA can be found, for instance, in Dunkin‐Hartley guinea pigs and primates. These species typically have a thicker subchondral plate^16^, ^17^, ^18^ with increased density and reduced porosity as well as increased trabecular bone volume fraction, anisotropy, plate‐like shape, and transverse orientation. These skeletal changes are thought to precede cartilage degeneration.^18^

In human (aging), OA a clinical hallmark is increased bone mineral density.^19^, ^20^ Histologically, it has been shown that in OA bone formation and volume are increased in subchondral trabecular bone with little resorption,^21^ whereas presence of resorption pits and microtrabeculae in human OA samples have also been reported.^22^ Changes in trabecular bone microstructure seem to follow a general pattern in various skeletal site: Tibia,^23^, ^24^, ^25^, ^26^, ^27^ femoral head,^28^ and neck.^29^ These changes include increase in trabecular bone volume fraction, thickness and reduced separation, whereas the overall trabeculae shape becomes rather plate‐like.^23^, ^24^, ^25^, ^26^, ^27^, ^28^, ^29^ Moreover, altered trabecular orientation and increased anisotropy have often been reported,^29^ though this effect has not always been obvious.^25^, ^28^ This controversy promotes the need for evaluating variations of local patterns in skeletal structures using alternative methods to better understand the bone changes in OA.^25^, ^28^, ^30^

In previous studies, the subchondral plate in OA has been reported to be thicker and more porous.^22^, ^31^ To our knowledge, there is no study reporting the actual relationship between histological disease severity and subchondral bone plate modifications analyzed volumetrically using human sample material. Moreover, calcified cartilage has been shown to increase its thickness with OA process^32^ but it becomes virtually absent in late OA.^22^, ^32^ Despite increasing evidence that the subchondral bone would contribute to the pathogenesis of OA,^4^, ^5^, ^6^, ^7^ most diagnostic methods are still focused mainly on cartilage degeneration and erosion, and skeletal changes are acknowledged only at late stages. Therefore, we hypothesized that subchondral bone changes occur in parallel with cartilage degeneration. To test this hypothesis, subchondral bone changes in both trabecular bone and subchondral plate were evaluated as a function of disease severity, evaluated by OARSI grading system.^33^Subchondral bone changes were analyzed by two methods: (1) traditional morphological analyses in whole tissue volume, and (2) analyses of local bone patterns using a volumetric local binary patterns (LBP)‐based analysis, which is a promising method to study distribution, orientation, continuity, and complexity of local structures.

## MATERIALS AND METHODS

### Human Osteochondral Samples

Tibial plateaus were collected from 14 late OA‐patients (age 76 ± 9 years: 2 males and 12 females) treated with total knee arthroplasty at Oulu University Hospital, Finland. Sample collection and the study protocol was approved by the Ethical Committee of the Northern Ostrobothnia Hospital District, Oulu, Finland (191/2000). Altogether, 25 osteochondral samples were prepared from tibial plateaus from areas visually classified into three categories in terms of macroscopical degeneration of the articular cartilage: (1) intact cartilage (*N* *= *11), (2) moderate cartilage erosion (*N* *= *7), and (3) partly or fully exposed subchondral bone (*N* *= *7). Cylindrical samples with a diameter of 6 mm were prepared from all macroscopic visual grades.

### Imaging and Evaluation of Structural Parameters

Samples were stored in phosphate‐buffered saline (PBS) for micro‐computed tomography (μCT) imaging. Osteochondral cylinders were scanned with a μCT device at isotropic 27.8 μm voxel size (Skyscan 1172, Bruker microCT, Kontich, Belgium). Images were reconstructed and analyzed with manufacturer provided software. The subchondral plate was manually segmented from the trabecular bone. Both trabecular and cortical plate compartments were analyzed separately. One sample did not have any trabecular bone and could not be included in the analyses. Images were pre‐thresholded to 60 and remaining pixels were binarized with adaptive thresholding using the mean of minimum and maximum values within a 5 pixel radius window. After binarization, images were subjected to morphological analyses and the outcome variables are described in ref.^34^

### The Volumetric Local Binary Patterns Method

In this study, a new alternative method, known as LBP^35^ was used for analysis of local bone changes as a function of histological OA grade. Briefly, in the LBP method, the neighborhood of a center pixel is evaluated for occurrences of equal or higher gray level values than in the center pixel. For each center pixel, a specific local pattern is then determined based on the locations of these occurrences which are called markers. The LBP method was applied to every pixel with at least one neighbor with a higher value than a fixed threshold in order to capture only information about the bone and avoid non‐relevant data from empty space.

LBP‐based analysis was performed with a custom‐made algorithm (MATLAB version R2013b, MathWorks, Natick, MA). The LBP method used here is an improved version of the one reported in the literature.^36^ While the previous analysis was performed in 2D, the method used here was developed to perform 3D volumetric analyses. For this purpose, instead of fitting 8 neighbors on a circle within the studied slice, a total of 26 neighbors were fitted on a sphere (Fig. S1) within multiple slices, the studied pixel being the center of the sphere. The gray‐level value of the neighbors was calculated by interpolation of the voxels values surrounding them. Histograms of patterns distribution were obtained from the analysis and the entropy of the local patterns was evaluated as well as the amount of different local patterns obtained. The histograms were converted to obtain the occurrences of each specific pattern as a percentage of all the patterns recognized within a VOI. The entropy of local patterns was calculated as follows:

(1) $$E=-\sum_{i}P_{i}\log_{2}(P_{i})$$

where *P_i_* contains the count of a specific local pattern *i* occurring in the image. If an image contains only one local pattern, the entropy of the patterns within the image is zero. The entropy here describes the randomness of local patterns in the volume of interest.

The elevation angle of each pattern was calculated using principal component analysis. The elevation is the angle along the distal‐proximal axis, representing the alignment of patterns toward the cartilage surface. The patterns without consistent orientation were ignored in the analysis, the following cases were considered as such: Not enough markers (<3), too many markers (>24), or when markers were located a further distance from the “centroid” than non‐markers. As the μCT imaging acquisition protocol did not allow maintaining the same reference orientation on the other plans, the azimuth angle was ignored in the analysis.

Both the entropy and the homogeneity of the elevation were calculated to describe spatial distribution of the elevation angles and to provide an indication on continuity of the structures. An angle‐level co‐occurrence matrix (ALCM) was first calculated with volumetric rotational invariance to assess the spatial distribution of the elevation angle. The homogeneity of the elevation was then calculated from the ALCM as such:

(2) $$H_{elevation}=\sum_{i,j}\frac{ALCM(i,j)}{1+|i-j|}$$

where each element (*i*,*j*) in the ALCM is the sum of the occurrences that a voxel with elevation *i* has a voxel with elevation *j* in its vicinity (one voxel‐size radius). Eventually, a high value of homogeneity of the elevation corresponds to data with consecutive voxels with similar elevation angles.

### Histopathology

**Figure 1:**
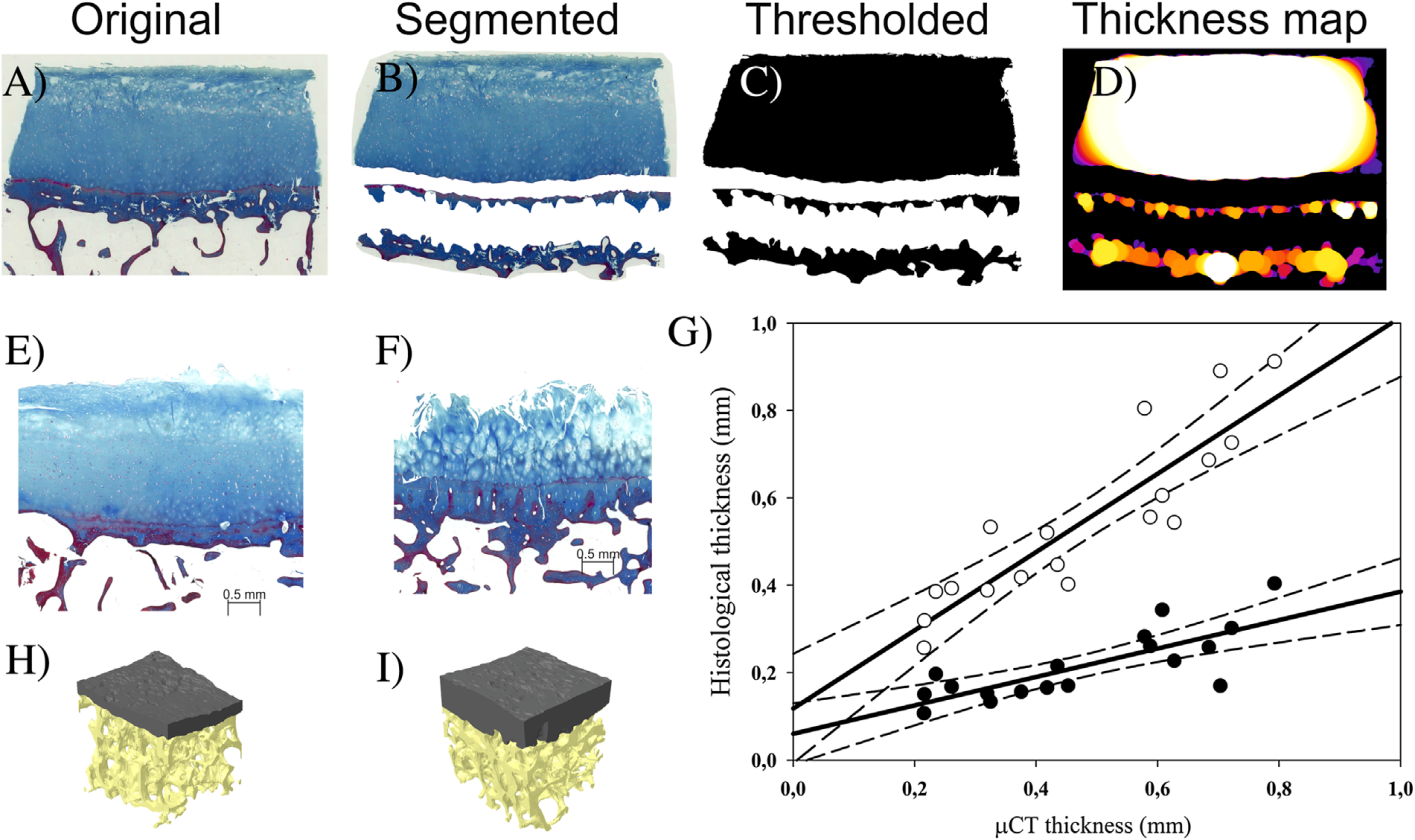
Analysis of joint tissue morphologies was performed from Masson's trichrome stained sections. From original images (A) cartilage, calcified cartilage and subchondral bone were manually segmented (B) followed by thresholding and procedure to remove closed pores to fill cell lacunae (C). These images were then subjected for thickness analyses (D). Regression analysis between μCT and histological thicknesses (G) shows best correlation to total thickness of mineralized structures (open circles), instead of subchondral bone alone (black circles). Finally, a comparison between reconstructed 3D models of analyzed bones, with OARSI grade of 1.6 (H) and other with OARSI grade of 4.1 (I) with respective histological images (E and F). In 3D models, subchondral plate is colored as gray and trabecular bone as yellow.

After μCT imaging, cylinders were formalin‐fixed and decalcified in EDTA. Paraffin‐embedded blocks were sectioned to 5 μm and stained with Safranin O and Masson's trichrome. OARSI grading^33^ was conducted with Safranin O stained sections. Each sample was graded from three sections by three independent evaluators (MF, OA, VT). The average grade from three evaluators was used as the final OARSI grade for further analyses. Samples with Masson's trichrome staining were scanned with a microscope slide scanner (Pannoramic 250F, 3DHISTECH, Budapest, Hungary) and images were exported to Fiji, where cartilage, subchondral plate, and calcified cartilage were manually segmented and their thicknesses were measured using a BoneJ plug‐in (Fig. 1A–D).^37^

### Statistical Analysis

Statistical analyses were performed using SPSS software (version 20.0; SPSS, Chicago, IL). To test precision manual segmentation was repeated for one sample 10 times and coefficient of variations (CV%) were calculated for morphometric parameters. Exploratory data analysis was performed by plotting structural parameters against OARSI grade and visualizing each TKR patient with specific markers. Although there is a large variation within a patient, possible effect of pseudoreplicates was removed by equally weighting samples from the same donor (*N* = 13 trabecular bone, *N* = 14 subchondral bone plate) for correlation analysis. Pearson's linear correlation coefficients were calculated between the OARSI grade and the parameters derived from both traditional structural analysis and the LBP method. To evaluate which structures were included in the μCT‐based plate thickness, these values were correlated against the histologically measured calcified cartilage, subchondral plate thickness, and their sum.

## RESULTS

All knees had large internal variation in OARSI grades depending on the site. The OARSI grades varied from 0.9 to 6.3 (mean ± SD = 3.5 ± 1.7). High precision was noted for both histological and μCT‐derived morphological parameters as indicated by CV% ranges 0.81–3.19% and 0.21–3.80%, respectively (Tables S1 and S2). Results from histological sections (Fig. S2) showed a thicker subchondral bone plate with higher OARSI grade (*R* = 0.67, *p* = 0.025). On other hand, cartilage thickness showed only moderate negative correlation (*R* = −0.57, *p* = 0.065) and there was no correlation with calcified cartilage thickness (*R* = −0.12, *p* = 0.720) when linked to OARSI grade. However, the total thickness of mineralized structures, calculated as the sum of subchondral plate and calcified cartilage thicknesses, showed even stronger correlation to OARSI grade (*R* = 0.83, *p* = 0.002). The subchondral bone plate thickness measured from histology was linearly correlated to the one derived from μCT (Fig. 1, *R* = 0.88, *p* < 0.001), the latter one being twice as thick. However, if the total thickness of mineralized structures from histological sections were compared against μCT‐derived plate thickness, μCT gave slightly smaller values, and the correlation (*R* = 0.98, *p* < 0.001) was stronger.

**Figure 2:**
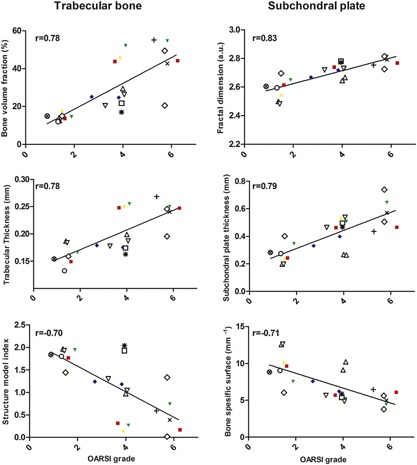
Right: Subchondral bone plate fractal dimension, thickness, and specific bone surface presented as a function OARSI grade. Left: Trabecular bone volume fraction, number, and entropy of local patterns as a function OARSI grade. Samples from each donor are plotted with different markers demonstrating larger variation of OARSI grades and μCT derived parameters within each tibia.

The majority of the structural and texture bone parameters showed strong correlations with OARSI grades (Table 1 and Fig. 2). The subchondral plate thickness and fractal dimension correlated positively with OARSI grade (*R* = 0.79 and *R* = 0.83, respectively) with decreasing bone specific surface (*R* = −0.71). The trabecular bone volume fraction increased with OARSI grade (*R* = 0.78) with decreasing bone specific surface (*R* = −0.79). Also the trabecular number (*R* = 0.80) and thickness (*R* = 0.78) increased, while their separation decreased (*R* = −0.74) leading to increased connectivity of trabecular network as indicated by increasing connectivity density (*R* = 0.73) and decreasing trabecular pattern factor (*R* = −0.78) with disease severity. Furthermore, trabeculae shape became more plate‐like as indicated by a negative correlation between structure model index and OARSI grade (*R* = −0.70). Conventional morphological analysis showed only moderately lower anisotropy (*R* = −0.47) with OARSI grade.

**Table 1:** Pearson Correlation Coefficients Between Subchondral Plate/Trabecular Bone Structural Parameters and OARSI Grade

Subchondral Plate Parameter	Correlation With OARSI Grade
Thickness (mm)	0.79**
Specific bone surface (1/mm)	−0.71* (*p* = 0.006)
Fractal dimension (a.u.)	0.83**
Trabecular Bone	Correlation With OARSI Grade
Bone volume fraction (%)	0.78* (*p* = 0.002)
Specific bone surface (1/mm)	−0.79**
Trabecular number (1/mm)	0.80**
Trabecular thickness (mm)	0.78* (*p* = 0.002)
Trabecular separation (mm)	−0.74* (*p* = 0.004)
Connectivity (a.u.)	0.17 (*p* = 0.58)
Connectivity density (1/mm^3^)	0.73* (*p* = 0.005)
Structure model index (a.u.)	−0.70* (*p* = 0.008)
Trabecular pattern factor (1/mm)	−0.78* (*p* = 0.002)
Degree of anisotropy (a.u.)	−0.47 (*p* = 0.11)
Fractal dimension (a.u.)	0.83**

a.u., arbitrary unit. *p < 0.05; **p < 0.001

**Figure 3:**
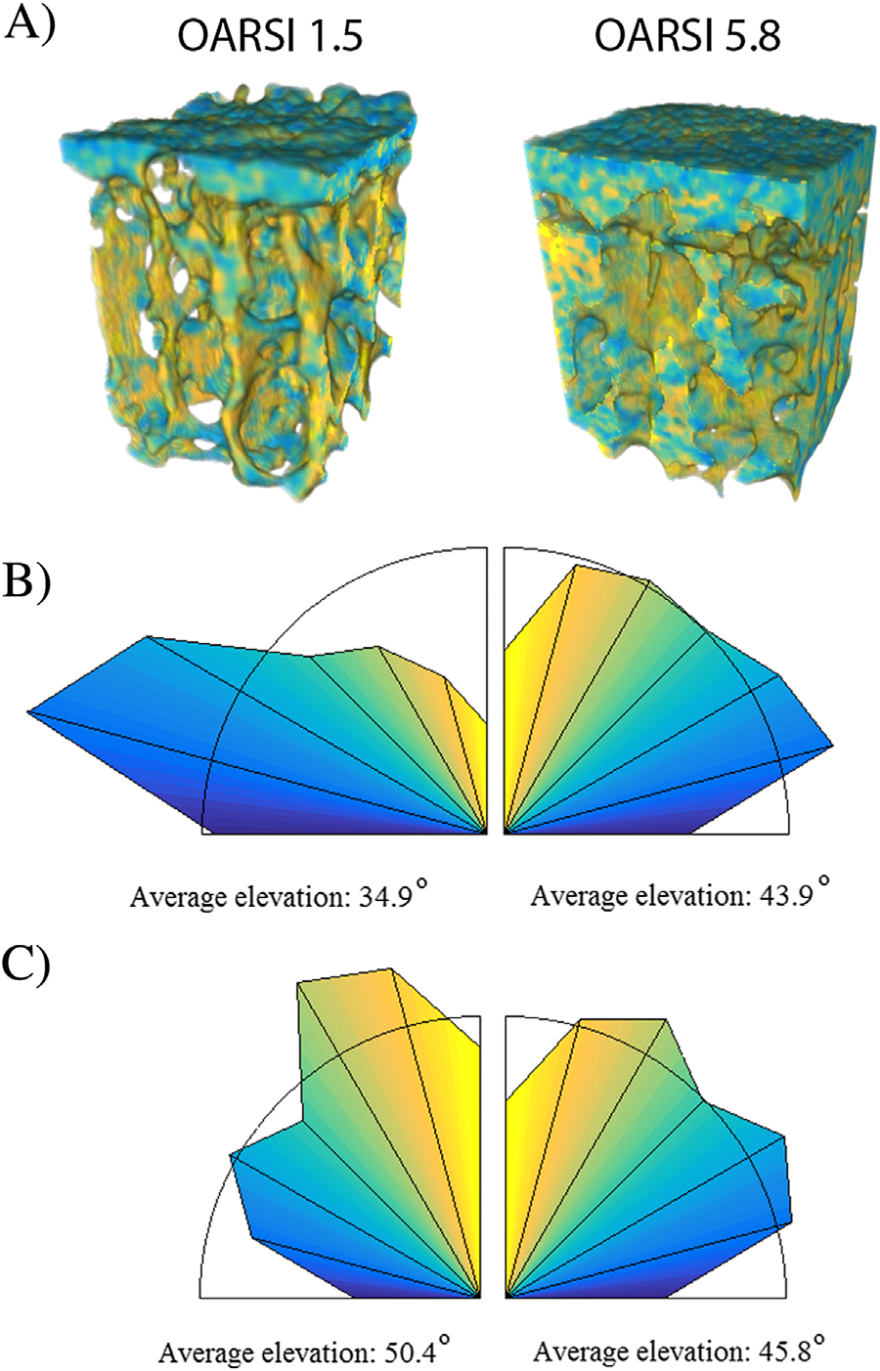
Visualization of color coded elevation angles from 3D local binary patterns (LBP) analysis from samples with OARSI grades of 1.5 and 5.8 (A). Normalized distribution of elevation angles shows that with increasing OARSI grade patterns are rather oriented longitudinal (yellow) in subchondral plate (B) and transverse (blue) in trabecular bone (C).

The novel 3D LPB analysis showed (Table 2) that in both the subchondral plate and trabecular bone compartments, a higher OARSI grade corresponded to a higher number of different patterns (*R* = 0.89 and *R* = 0.69, respectively) and an increase in the entropy of patterns (*R* = 0.80 and *R* = 0.83, respectively). Samples with high OARSI grades presented a stronger proportion of patterns oriented longitudinally in subchondral plate and transversally in trabecular bone (Fig. 3) than samples with lower OARSI grades. The homogeneity of angle elevation was also negatively correlated with OARSI grade in subchondral plate (*R* = −0.80) as well as trabecular bone (*R* = −0.79) while elevation entropy increased (*R* = 0.79 and *R* = 0.69, respectively) with OARSI grade. These results indicate discontinuity in the orientations within consecutive patterns, suggesting sharper angles in the architectures and eventually higher complexity of the structures in general.

**Table 2:** Pearson Correlation Coefficients Between Subchondral Plate/Trabecular Bone LBP Parameters and OARSI Grade

Subchondral Plate	Correlation With OARSI Grade
LBP: Average number of markers (a.u.)	0.73* (*p* = 0.005)
LBP: Number of different patterns (a.u.)	0.89***
LBP: Patterns entropy (a.u.)	0.80**
LBP: Elevation entropy (a.u.)	0.79**
LPB: Homogeneity of elevation (a.u.)	−0.80**
LPB: Average elevation (°)	0.82***
Trabecular Bone	Correlation With OARSI Grade
LBP: Average number of markers (a.u.)	0.86***
LBP: Number of different patterns (a.u.)	0.69* (*p* = 0.01)
LBP: Patterns entropy (a.u.)	0.83***
LBP: Elevation entropy (a.u.)	0.69* (*p* = 0.009)
LPB: Homogeneity of elevation (a.u.)	−0.79* (*p* = 0.002)
LPB: Average elevation (°)	−0.53 (*p* = 0.06)

LBP, local binary pattern analysis; a.u., arbitrary unit; *p < 0.05; **p < 0.001; ***p < 0.0001

## DISCUSSION

It is known that subchondral bone becomes more sclerotic in OA,^2^ but there is little evidence where the earliest microstructural changes in OA actually start—within cartilage or subchondral bone. Although this long standing question remains to be further elucidated, our results indicate that subchondral bone modifications are strongly coupled to articular cartilage degeneration already at the lowest OARSI grades. Morphometric parameters of subchondral plate and trabecular bone were evaluated separately due to the specific structure and functions leading to possibly different adaptation to OA in these compartments.^38^ Our results indicate that OA‐related alterations are present in both.

Our results showed higher trabecular bone volume fraction with OA due to higher trabecular number and thickness and lower trabecular separation. In this study, one of the calculated morphological parameters was SMI. This metric gives an estimation of the shape of the trabecular structures, from plate to rod‐like. Here, we observed a negative correlation between SMI and OARSI grade, corresponding to a plate‐like appearance of trabeculae in advanced OA. This finding was expected based on previous morphometric guinea pig model of spontaneous OA^17^, ^18^ and human studies.^23^, ^24^, ^25^, ^26^, ^27^, ^28^, ^29^ As an adaptation to OA, trabecular bone may increase its stiffness and strength^39^, ^40^, ^41^ by changing its structure to resist higher loading due to cartilage loss.

For both the subchondral plate and trabecular bone, the specific bone surface decreased with OA. This parameter corresponds to the ratio of bone surface by volume, suggesting that the bone surfaces become more uniform in trabecular bone and smoother in subchondral plate with increasing OARSI grade. In a recent study,^14^ a large variation in subchondral plate thickness was observed within healthy and OA knees, with a tendency for the plate to become thicker with disease progression. As expected, the subchondral bone plate became thicker with higher OARSI grades and this relationship was surprisingly linear. To further validate this observation, thickness of calcified cartilage and subchondral plate from the Masson's trichrome stained histological sections were measured. These results revealed that μCT‐derived subchondral plate thickness also includes calcified cartilage. We further tested if histological subchondral plate or calcified cartilage thickness correlate with OARSI grade. Parallel to μCT subchondral plate thickness, histological subchondral plate showed positive but weaker correlation to OARSI grade, while there was no linear correlation to the calcified cartilage thickness. This lack of correlation could be expected as there is denudation of cartilaginous structures at OARSI grades >5. It could be that the earliest change in OA is the advancing tidemark, which makes the actual hyaline cartilage thinner and allows thickening and stiffening of subchondral plate, thereby increasing stress in the cartilage to pathological level. In OA, increased osteoclastic resorption in subchondral bone can extend over the calcified cartilage,^42^ increasing the porosity of the subchondral plate^43^ and its hydraulic conductance.^32^ Also in post‐traumatic OA models, accelerated bone remodeling has been observed as indicated by an increased number of osteoclasts and osteoid in subchondral bone, increasing its permeability (angiogenesis).^14^ Nevertheless, our results showed the best correlation between thickness of mineralized structures and OARSI only when subchondral plate and calcified cartilage where accounted for. However, the role of tidemark in OA pathogenesis is still unclear and should be further considered in future studies.^44^

These results highlight the potential of evaluating OA severity from mineralized structures. In longitudinal in vivo studies, this could be achieved either by following changes in calcified cartilage using ultrashort echo time magnetic resonance imaging^45^ or evaluating subchondral plate and trabecular bone properties from high resolution clinical CT. The latter may become even more of an attractive option when combined with novel image processing tools such as 3D LBP.

In this study, the feasibility of applying a novel 3D LBP image analysis method related to local bone patterns in μCT scans was evaluated. By using 3D LBP‐based method, it was possible to derive information on distribution, orientation, and complexity of patterns. The primary advantage of LBP is its low sensitivity for monotonic grayscale variations.^46^ This is important because LBP may be ideally suited for clinical CT or extremity CT images where beam hardening and partial volume effect can reduce the variation in gray‐levels to an extent that individual structures cannot be accurately segmented using global or even adaptive thresholds.

To date, the LBP method has been applied solely in 2D to analyze local bone structures in OA.^36^, ^47^ As noted earlier, entropy of local patterns is a measure of the randomness of the different bone patterns appearing in the region or volume of interest. Recently, it has been shown that the entropy of local patterns was related to OA severity assessed via K‐L grading scale,^47^ which parallels our findings. Also LBP‐derived structural local bone properties identified in this study are in accordance with previous fractal‐based studies showing significant differences between controls and patients with OA.^48^

The current study showed that the elevation of patterns with increasing disease severity indicated that the subchondral bone plate and trabecular bone have different characteristics, which is expected since these compartments have different morphology, physiology and mechanical properties.^4^ The higher complexity and discontinuity of the local consecutive patterns (in terms of orientations) in both compartments is directly related to altered bone structure as more connections between the bony structures are created with advancing OA.^29^ In the subchondral bone plate, the appearance of non‐uniform patterns could indicate altered porosity and tissue heterogeneity. In trabecular bone, the degree of anisotropy was lower with higher OARSI grades as previously suggested.^25^, ^28^, ^29^ The novel 3D LBP method clearly demonstrated higher entropy and a rather transverse orientation (Fig. 3). This altered orientation fits well with the early proposal by Radin and Rose about adaptation of subchondral bone to shear stress.^49^

This study highlights the importance of subchondral bone changes in human OA. Most importantly, these results indicate that changes in articular cartilage and subchondral bone are likely to be coupled, at least in this sample set. Therefore, based on this data, the cartilage‐driven initiation of OA cannot be supported. However, it should be mentioned that according to current understanding, OA is not just a single disease but instead a group of diseases with different phenotypes.^50^ It may be that the current samples represents an ageing phenotype, where both cartilage and bone changes occur with relatively the same speed. On the other hand, post‐traumatic OA, for example, could be a phenotype starting from the articular cartilage defect, leading to subsequent subchondral bone changes. However, at this stage, this can be only speculated and more research is needed to understand the etiopathology of OA and its different phenotypes.

A major strength of this exploratory study pertains in comprehensive analysis of bone and cartilage using histological methods as well as μCT analyses with both conventional and novel LBP‐based methods. Furthermore, as this study focused on smaller volumes directly under histologically graded area, the link between bone morphometric parameters and OARSI grade was highly linear. A major limitation, however, remains concerning the tissue samples from late‐OA patients scheduled for knee surgery. Given their nature, it cannot be known for sure whether the samples with low OARSI grades are truly representative of early OA bone and cartilage. To fully confirm this, a larger size of human samples from both symptomatic and asymptomatic donors is required.

In summary, results obtained from volumetric standard bone parameters, as well as parameters from local analysis of bone patterns, were complementary. The high correlation between the OARSI grade and the subchondral plate thickness and the trabecular bone volume fraction indicate that bone sclerosis and cartilage degeneration are coupled. Furthermore, the novel LBP‐based method is a potential tool for evaluating both quantitative and qualitative changes from low‐resolution clinical images. Altogether, this study demonstrated the possibility to diagnose severity of OA using bone structural analysis, which eventually could be assessed in clinical settings.

## AUTHORS’ CONTRIBUTIONS

MF, JT, MTN, PL, SS contributed to study design. MAJF, OA, VT, JR, SK, MV, PL contributed to data collection. MAJF, JT, SK, KP contributed to data analysis. MAJF, JT, KP, PL, SS contributed to data interpretation. MAJF, JT contributed to manuscript preparation. All authors contributed to revising and approval of manuscript content. MF takes responsibility for the integrity of the work.

## Supporting information

Additional supporting information may be found in the online version of this article.

**Figure S1**. Demonstration of the LBP‐based method.
**Figure S2**. Correlation between OARSI grade and histologically defines thicknesses of different osteochondral tissues: In upper row thicknesses of cartilage and calcified cartilage on left and right respectively.
**Table S1**. Coefficient of variations for subchondral plate and trabecular bone structural parameters.
**Table S2**. Pearson correlation coefficients between cartilage, calcified cartilage and subchondral plate thickness from histological images, and OARSI grade and coefficient of variations for thicknesses analyzed from histological images
