# Supplementary material for: Association between subchondral bone structure and osteoarthritis histopathological grade
Source: J Orthop Res. 2016 Jun 22;35(4):785–92. doi: 10.1002/jor.23312 (PMC5412847; doi:10.1002/jor.23312)
Supplement: Supplementary file 1 — Figure S1. Demonstration of the LBP‐based method. Figure S2. Correlation between OARSI grade and histologically defines thicknesses of different osteochondral tissues: In upper row thicknesses of cartilage and calcified cartilage on left and right respectively. Table S1. Coefficient of variations for subchondral plate and trabecular bone structural parameters. Table S2. Pearson correlation coefficients between cartilage, calcified cartilage and subchondral plate thickness from histological images, and OARSI grade and coefficient of variations for thicknesses analyzed from histological images [file JOR-35-785-s001.docx]

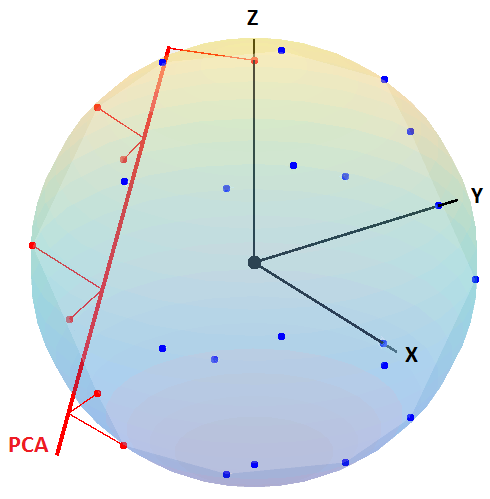


**Figure S1.** Demonstration of the LBP-based method. Red dots represent neighbors that have been considered in the pattern and blue ones are the excluded ones. The neighbors (in red and blue) are fitted on a sphere and the PCA is applied solely on valid markers (in red) to extract the orientation of the pattern.

**
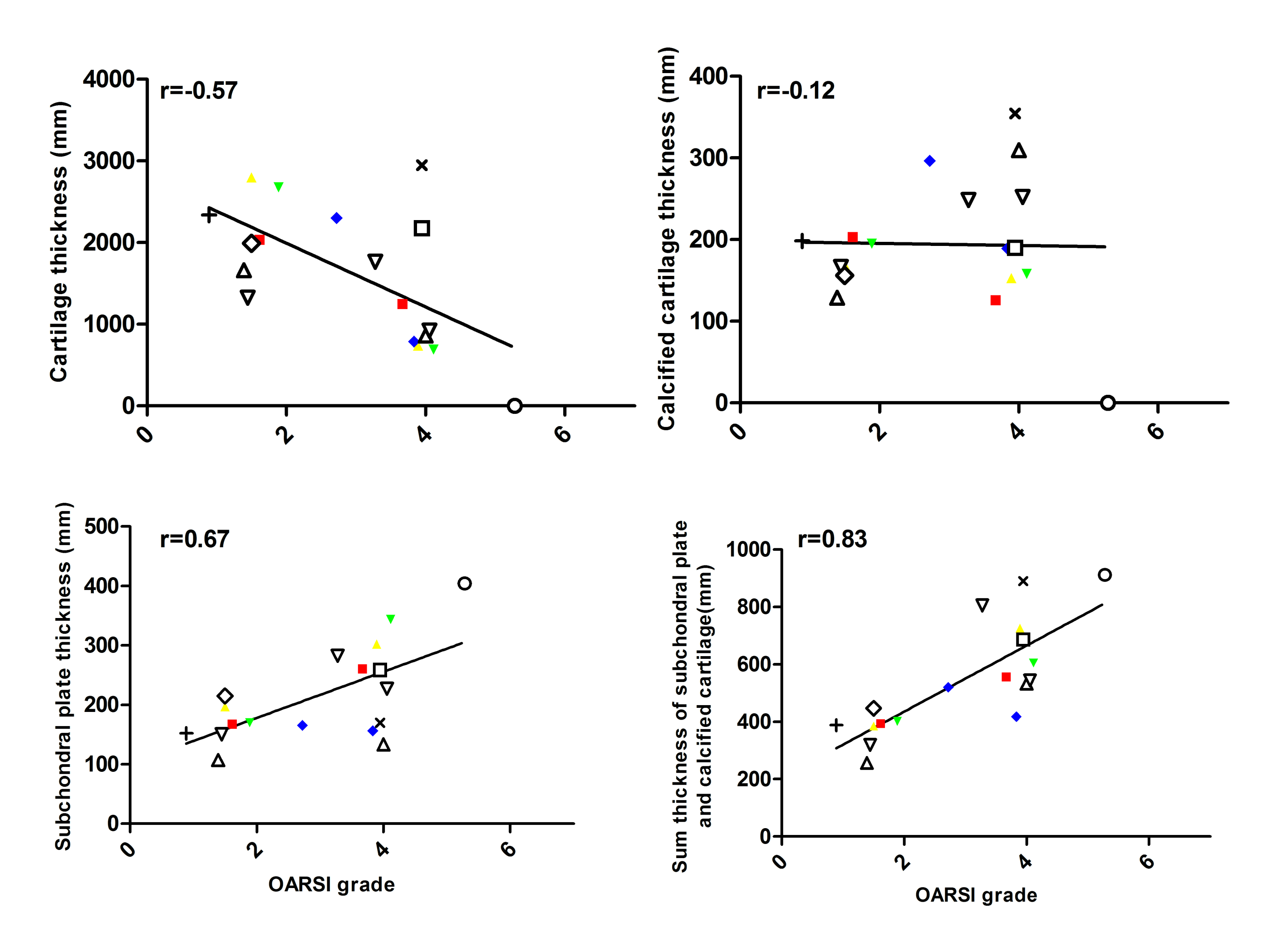
**

**Figure S2.** Correlation between OARSI grade and histologically defines thicknesses of different osteochondral tissues: In upper row thicknesses of cartilage and calcified cartilage on left and right respectively. Lower row: subchondral plate thickness on left and on right sum thickness of subchondral plate and calcified cartilage.

**Supplementary table 1:** Coefficient of variations for subchondral plate and trabecular bone structural parameters.

| **Subchondral plate parameter** | **CV%** |
| --- | --- |
| **Thickness (mm)** | 0.96% |
| **Specific bone surface (1/mm)** | 0.53% |
| **Fractal dimension (a.u.)** | 0.55% |
| **Trabecular bone** | **CV%** |
| **Bone volume fraction (%)** | 1.07% |
| **Specific bone surface (1/mm)** | 0.36% |
| **Trabecular number (1/mm)** | 1.02% |
| **Trabecular thickness (mm)** | 0.24% |
| **Trabecular separation (mm)** | 1.90% |
| **Connectivity (a.u.)** | 3.80% |
| **Connectivity density (1/mm^3^)** | 3.71% |
| **Structure model index (a.u.)** | 1.94% |
| **Trabecular pattern factor** | 2.67% |
| **Degree of anisotropy** | 1.57% |
| **Fractal dimension** | 0.21% |

* p<0.05; ** p<0.001

**Supplementary table 2:** Pearson correlation coefficients between cartilage, calcified cartilage and subchondral plate thickness from histological images, and OARSI grade and coefficient of variations for thicknesses analyzed from histological images.

| **Histological segmentation** | **Correlation with OARSI grade** | **CV%** |
| --- | --- | --- |
| **Cartilage Thickness (mm)** | -0.57 (p=0.065) | 1.49% |
| **Calcified Cartilage Thickness (mm)** | -0.12 (p=0.720) | 0.81% |
| **Subchondral bone Thickness (mm)** | 0.67* (p=0.025) | 3.19% |
| **Sum thickness of subchondral plate and calcified cartilage (mm)** | 0.83* (p=0.002) | 1.94% |

N=18, * p<0.05
